# Implications of back-and-forth motion and powerful propulsion for spirochetal invasion

**DOI:** 10.1038/s41598-020-70897-z

**Published:** 2020-08-18

**Authors:** Keigo Abe, Toshiki Kuribayashi, Kyosuke Takabe, Shuichi Nakamura

**Affiliations:** 1 Department of Applied Physics, Graduate School of Engineering, Tohoku University, 6-6-05 Aoba, Aoba-ku, Sendai, Miyagi 980-8579 Japan; 2 Present Address: Faculty of Life and Environmental Sciences, University of Tsukuba, 1-1-1 Tennodai, Tsukuba, Ibaraki 305-8572 Japan

**Keywords:** Biophysics, Microbiology

## Abstract

The spirochete *Leptospira* spp. can move in liquid and on a solid surface using two periplasmic flagella (PFs), and its motility is an essential virulence factor for the pathogenic species. Mammals are infected with the spirochete through the wounded dermis, which implies the importance of behaviors on the boundary with such viscoelastic milieu; however, the leptospiral pathogenicity involving motility remains unclear. We used a glass chamber containing a gel area adjoining the leptospiral suspension to resemble host dermis exposed to contaminated water and analyzed the motility of individual cells at the liquid-gel border. Insertion of one end of the cell body to the gel increased switching of the swimming direction. Moreover, the swimming force of *Leptospira* was also measured by trapping single cells using an optical tweezer. It was found that they can generate $\sim$ 17 pN of force, which is $\sim$ 30 times of the swimming force of *Escherichia coli*. The force-speed relationship suggested the load-dependent force enhancement and showed that the power (the work per unit time) for the propulsion is $\sim$ 3.1 × 10^–16^ W, which is two-order of magnitudes larger than the propulsive power of *E. coli*. The powerful and efficient propulsion of *Leptospira* using back-and-forth movements could facilitate their invasion.

## Introduction

Motility has been identified as a crucial virulence factor for pathogenic bacteria^1^. For example, a motility-deficient mutant of *Vibrio cholerae* is attenuated due to the decreased invasion efficiency of the epithelium^2^. In some flagellated bacteria, both motility and flagella are considered essential as an adhesin. For example, *Salmonella enterica* attaches to the host tissue via peritrichous flagella, which results in colonization and clinical outcomes^3^. Although spirochetes, such as *Borrelia burgdorferi* (the Lyme disease)^4^ and *Brachyspira hyodysenteriae* (swine dysentery)^5^, also utilize motility during infection, their flagella exist beneath the outer membrane, which is known as the periplasmic flagella (PFs), and spirochetal flagella are not directly involved in pathogenicity. Instead, the improvement of swimming ability^6^ and diverse adherence^7^ in viscoelastic environments is believed to be responsible for their colonization and dissemination within hosts.

**Figure 1:**
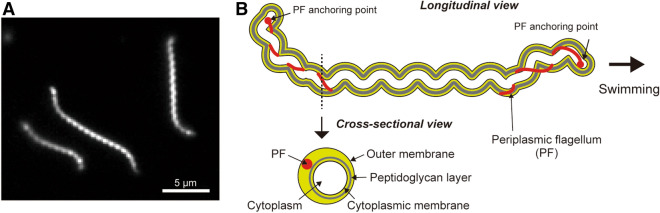
Morphology of *Leptospira*. **(A)** Dark-field micrograph of *Leptospira kobayashii*. **(B)** Schematic diagram of the cell structure of *Leptospira* spp.; a swimming cell exhibiting the spiral anterior end (right side of the diagram) and hook-shaped posterior end (left side) is illustrated.

The genus *Leptospira* is a member of spirochetes, and these pathogenic species have been found to cause a worldwide zoonosis known as leptospirosis. Pathogenic *Leptospira* cells are maintained in the proximal renal tubules of rodents as a reservoir. When the hosts urinate, they spread the spirochetes into the environment; as a result, many mammals, including humans, are percutaneously or transmucosally infected by contact with the contaminated soil and water^8,9^. *Leptospira* spp. have a right-handed spiral cell body and exhibit curvatures at both ends (Fig. 1A). Spirochetes can swim in liquid and crawl on surfaces using two PFs (one PF/cell end) (Fig. 1B). The morphology of the cell ends frequently changes between a spiral and a hook shape; and there is an asymmetric configuration of spiral and hook shapes at the anterior and posterior cell ends, respectively, that propels the cell unidirectionally (Fig. 1B)^10–13^. Similar to other motile species, the motility of *Leptospir*a spp. is closely related to their pathogenicity^14–16^, although how it contributes as a virulence factor in the spirochete remains to be unknown.

We explored one explanation for motility dependence in the pathogenicity of *Leptospira*. We showed that the spirochete increased the frequency of changes in the swimming direction with an elevated viscosity of polymer solutions, and the limitation of their net migrations could be involved in the accumulation of the spirochete in the epithelial mucosa in vivo^12^. In this study, we hypothesized that the phenomenon observed in these polymer solutions for percutaneous infection of *Leptospira* is significant, and examined their behaviors at the border of liquid and gel phases to mimic the skin dermis, which is exposed to contaminated environmental water. Furthermore, we expected that there would be a sizable propulsive force for the invasion and, thus, performed force measurements of swimming spirochetes using optical tweezers. Our experiments showed that there is an enhancement of swimming reversal when only one end of the cell body is inserted into the gel, and there is also a much larger swimming force of *Leptospira* than the known values of exoflagellated bacteria. These results suggest that powerful and efficient swimming with repeated trial-and-error allows *Leptospira* to obtain a smooth passage for penetration through the host dermis.

## Results

### Swimming reversal

**Figure 2:**
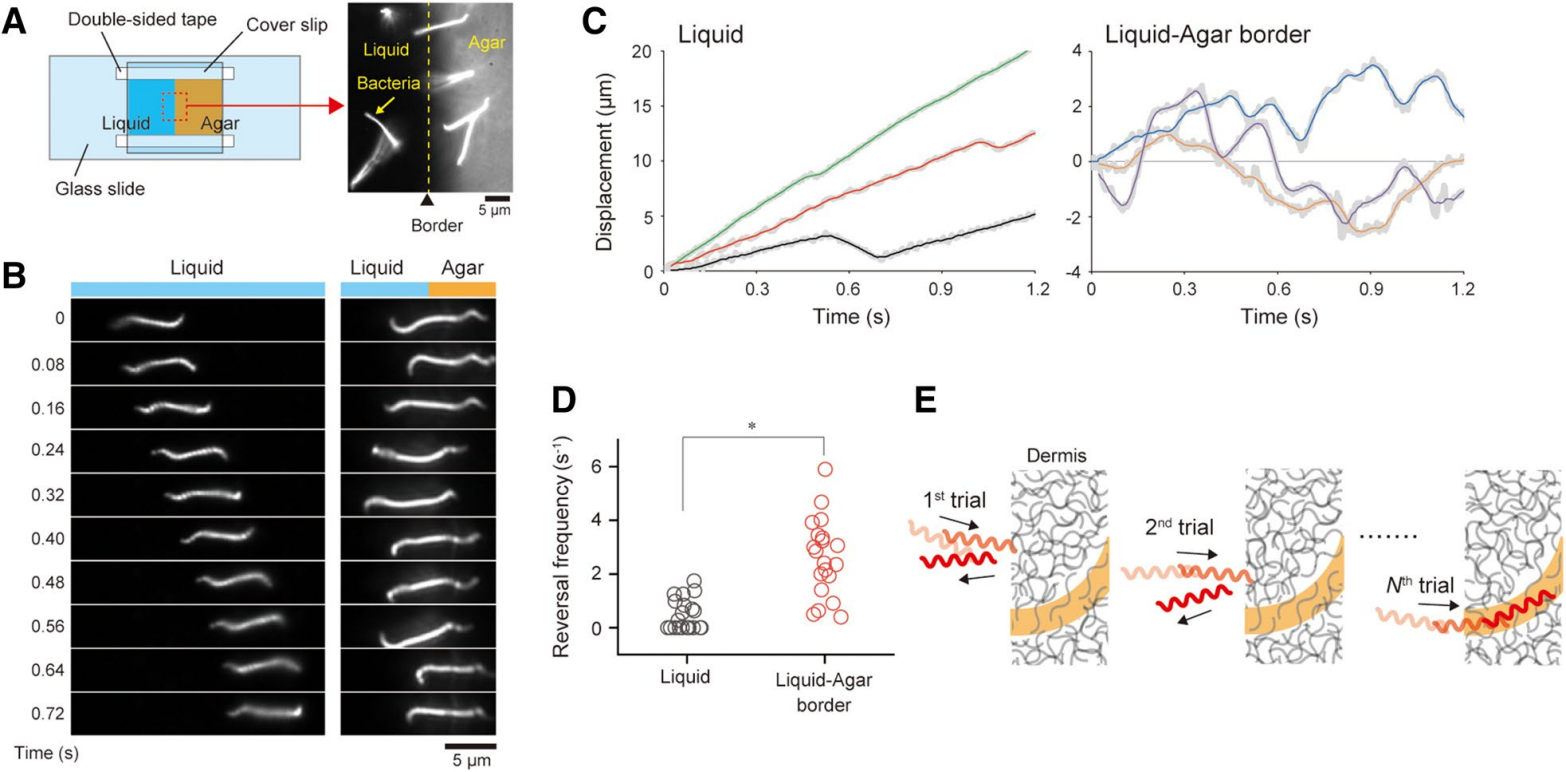
Swimming reversal. **(A)** Illustration of a flow chamber in which liquid and agar areas are contiguous. The microscopic image shows the liquid-agar border. Bacteria swam in liquid at the beginning of the experiment and then penetrated the agar area. **(B)** Time courses of the cell-movements observed in liquid (left) and at the liquid-agar border (right). **(C)** Displacements of individual cells. Example data obtained from three *L. kobayashii* cells are shown, each in liquid and at the border. Thick gray lines indicate raw data obtained by determining cell positions at 4 ms intervals, and thin colored lines are the results of a 12-data-point moving average. **(D)** Comparison of the reversal frequency determined from the displacement data of *L. kobayashii* cells. The results of liquid highlight no reversals occurred during observation. Mann-Whitney U test showed significant difference (*P < 0.05); n = 24 cells for liquid, and n = 20 cells for the border. **(E)** A plausible contribution of frequent swimming reversals for invasion. Back-and-forth movements give the cell chance to find a more accessible route in heterogeneous dermis structure (orange). Video 1 demonstrates “trial-and-error” of the *L. kobayashii* cell at the liquid-agar border by swimming reversals.

We observed spirochetes using a flow chamber containing adjoining liquid medium and agar to examine the behaviors of *Leptospira* during penetration of viscoelastic environments (Fig. 2A). The saprophytic species *Leptospira kobayashii* was used in the majority of the experiments. *Leptospira* cells show relatively smooth swimming in this liquid (Fig. 2B left and Fig. 2C left). In contrast, when one end of the cell body becomes inserted in the agar, the cells were seen to frequently change direction (Fig. 2B right and Fig. 2C right). The significant difference in reversal frequencies between cells in liquid and at the liquid-agar border is shown in Fig. 2D. Video 1 shows a *Leptospira* cell successfully penetrating agar after several back-and-forth movements. The enhancement of swimming reversal at the liquid-agar border was also observed in different species of *Leptospira* (Video 2 for the pathogenic species *L. interrogans* and Video 3 for the saprophyte *L. biflexa*), which suggests that the phenomenon is shared among genera. For pathogenic species, exploring more accessible routes in the dermis, such as structurally disturbed parts (due to injury), and using a “trial-and-error” method could determine the involvement in percutaneous invasion (Fig. 2E).

### Force measurement for swimming *Leptospira*

**Figure 3:**
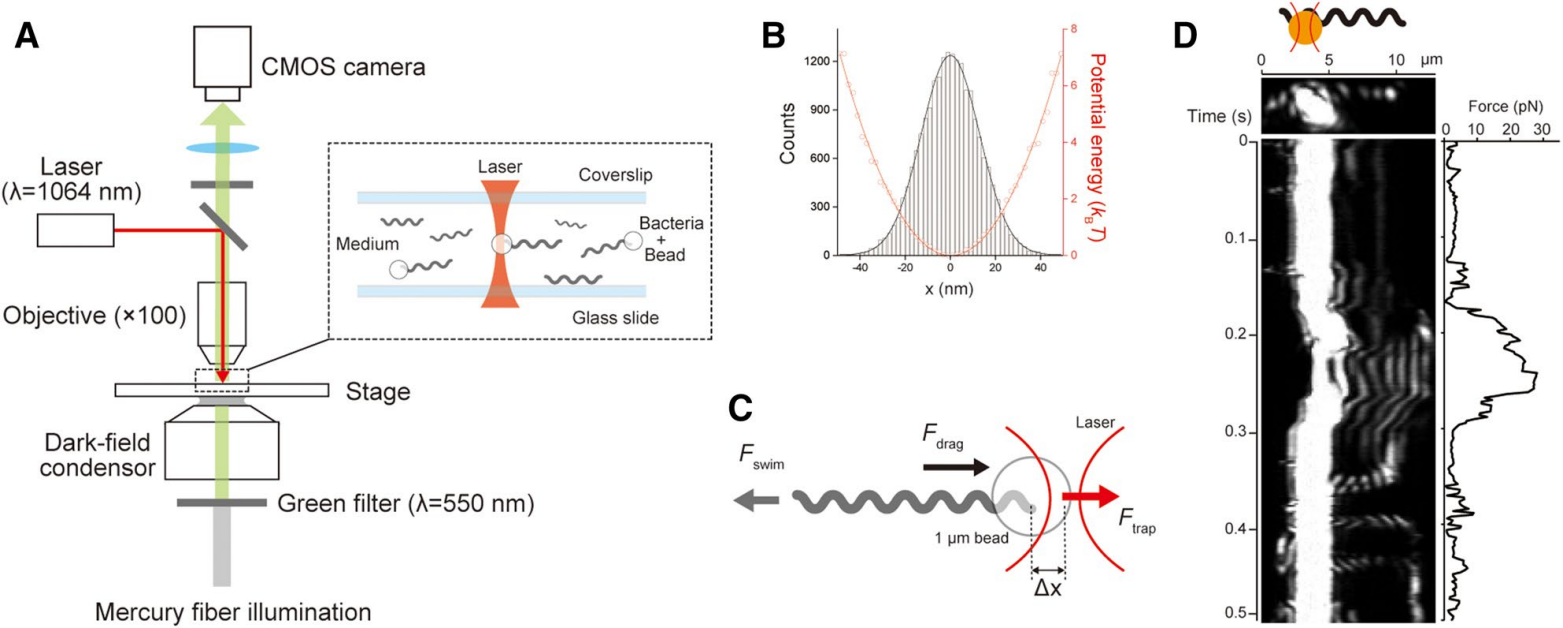
Swimming force measurement. **(A)** A dark-field microscope equipped with an optical tweezer. **(B)** The distribution of the positions of a 1-μm bead trapped by optical tweezers (histogram) and the estimated potential profile (red circles). The black and red lines indicate the results of the curve fitting by the Gaussian distribution and harmonic function, respectively. Spring constants were measured in each chamber, and 16–36 pN/μm were used. **(C)** Force balance in a swimming cell trapped by optical tweezer. See “Methods” for details. **(D)** The time course of the bead position is attached to a leptospiral cell and is trapped by an optical tweezer. The left upper schematic represents a trapped *L. kobayashii* via a 1 μm bead; the left middle and lower panels show a still image of a cell trapped via a bead and a kymograph showing bead movement with the leptospiral swimming, respectively. The right panel indicates the time course of the force estimated from the bead movement. See also Video 5.

**Figure 4:**
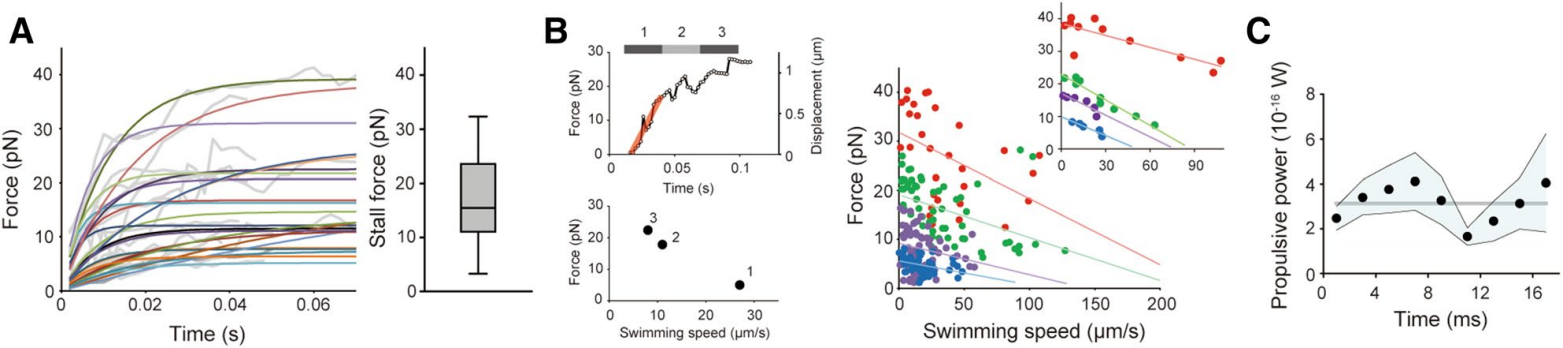
Swimming force. **(A)** Time courses of the swimming force obtained from 24 *L. kobayashii* cells. Gray and colored lines indicate the measured force curves and the results of exponential curve fitting, respectively. All of the force curves are shown in Supplementary Fig. S1 separately. The right panel shows the stall forces determined by the curve fitting; the boxes show the 25th (the bottom line), 50th (middle), and 75th (top) percentiles, and the vertical bars show the standard deviation. (**B**, left) Conversion from the force-time plot to the force-speed relationship, which is explained using an example trace extracted from *A*. The data plotted in the force-time plot was separated with an equal time interval, and the averaged forces of each bin were plotted against the average speeds determined by line fitting as shown in red in the upper panel. (right) The force-speed curve obtained from 24 cells were classified into four groups by stall force: <10 pN (blue), 10-20 pN (purple), 20-30 pN (green), and >30 pN (red). The colored lines are the regression lines fitted to each group. Example data extracted from each group are shown in the inset. **(C)** Time course of the power of the *Leptospira* swimming calculated by $Fv$. Black dots and the blue band indicate average values at each time point (n = 24 cells) and standard error, respectively. The horizontal gray line indicates the average power of the entire time course (3.1×10^-16^ W).

We trapped a microbead attached to swimming cells with optical tweezers in order to measure the swimming force of *Leptospira* (Fig. 3A, B). The labeling of *Leptospira* with antibody-coated microbeads was previously performed^17–19^ and showed that the attached microbeads move on the outer membrane because of the mobility of the targeted antigens and viscous drag exerted on the bead, i.e., the bead is retarded from cell movement by viscous drag. The spontaneous attachment of beads to the *Leptospira* cell surface without any linkers (nonspecific binding) was utilized in this experiment^19^, but bead movements were also observed (Video 4). To determine the force produced by unidirectional swimming, we measured cells with a bead at the posterior end of the cell body. Bead displacements were converted to swimming force ($F$) by considering the balance with trapping force (${F}_{trap}$) and drag force (${F}_{drag}$) exerted on the bead; $F={F}_{trap}+{F}_{drag}$ (Fig. 3C, D and Video 5). The swimming force increased with cell displacement and reached saturation when the cell became stalled by the restoring force of the laser trap. The stall force differed widely among measured cells, and the averaged force-time curve showed a stall force of 16.6 ± 2.2 pN (mean ± standard error; n = 24 cells) (Fig. 4A). A model experiment in which a tungsten coil, mimicking the shape of *Leptospira,* was rotated in a rotational magnetic field, showed that 2.2 pN of force is required for penetrating agar that resembles skin dermis^20^. Actual *Leptospira* cells could also produce a swimming force approximately eightfold higher than the previous model predicted. Furthermore, the swimming force of *Leptospira* is found to be $\sim$ 30 times greater than that of *E. coli* (~ 0.6 pN)^21^.

### Force–speed relationship

The relationship between force and speed was obtained from force-time plots, which showed that swimming forces linearly decreased as the swimming speed increased (Fig. 4B). The force–speed curves suggest that *Leptospira* can vary their propulsive force depending on the load exerted on the cell such as viscous drag and trapping forces. We calculated the work per unit time, $Fv$, for *Leptospira* propulsion using measured values (Fig. 4C). The propulsive power of *Leptospira* ranges from 1 × 10^−16^ to 7 × 10^−16^ W, which is two orders of magnitude greater than that of *E. coli* (5.8 × 10^−18^ W)^21^. These results suggest that *Leptospira* can produce a large force and maintain high efficiency when penetrating gel-like viscous materials.

## Discussion

We focused on the association of motility with the invasion of the *Leptospira* spirochete. Our biophysical experiments reveal that the back-and-forth movement of *Leptospira* is enhanced when only one of the cellular ends is exposed to a high viscosity medium and spirochetes can produce a much larger swimming force than exoflagellated bacteria. The trial-and-error behavior that permits the cell to find preferable entrances will be significant for infection through the dermis because heterogeneous fibrous structures may obstruct spirochetal invasion even though they can elicit high propulsion forces. The viscosity-dependent swimming reversal was observed in both pathogenic and nonpathogenic species, which implies that *Leptospires* can penetrate the dermis regardless of kinematic pathogenicity. However, since the motility of nonpathogenic *L. biflexa* is lost immediately after exposure to physiological osmotic conditions^22^, dissemination within the host is not deemed possible.

Previously, the enhancement of swimming reversal was observed in polymer solutions where the entire spirochetal body was exposed to changes in environmental viscosity or viscoelasticity^12^. However, we found that swimming reversal is increased by a minimal part of the cell body that is placed in the viscoelastic milieu. Because the torque for rotating the spiral body of *Leptospira* is produced by PFs residing at both cellular ends, it is expected that the two PFs should cooperatively rotate. In this context, our results suggest that some signal transduction between the two cell ends. Thus, one issue that remains unclear is the mechanism by which *Leptospires* sense a viscoelastic change at one end and rapidly transmit the mechanical signal to the other end (within < 1 s; see Video 6 for this rapid reversal). Coordinated rotations observed between *E. coli* flagellar motors depend on the diffusion of the phosphorylated chemotactic signaling protein CheY-P^23^. *Leptospira* spp. also possess several CheY homologs^24^. However, the formula $t={x}^{2}/D$ indicates that for CheY with a diffusion coefficient $D$ = 10 μm^2^/s^23^, there is an estimated 40 s (t) to diffuse $x$ = 20 μm (the approximate distance between the leptospiral motors). Therefore, rather than cytoplasmic signaling, the relatively stiff protoplasmic cylinder may be a medium for mechanical signal transduction^11,25^. Thus, elucidating the PF coordinated control mechanism warrants further investigation.

The measured swimming force of *Leptospira* is found to be $\sim$ 30 times greater than *E. coli*^21^. This ability to generate a higher force by the spirochete could be attributed to drag coefficients and high torque from the motor. Hydrodynamic studies of a low Reynolds number using resistive force theory showed that the drag force exerted on a spherical cell body with a spiral thin body, which rotates at ${\omega }_{\mathrm{Lep}}$ and translates at ${v}_{\mathrm{Lep}}$ in liquid, is calculated using ${F}_{\mathrm{Lep}}={\alpha }_{\mathrm{Lep}}{v}_{\mathrm{Lep}}+{\beta }_{\mathrm{Lep}}{\omega }_{\mathrm{Lep}}$, where ${\alpha }_{\mathrm{Lep}}$ and ${\beta }_{\mathrm{Lep}}$ are the drag coefficients for the spiral cell body^6,11,26^. Similarly, the drag force on externally flagellated bacteria with a spherical body and flagellum is given by ${F}_{\mathrm{Efb}}={\alpha }_{\mathrm{Efb}}{v}_{\mathrm{Efb}}+{\beta }_{\mathrm{Ef}}{\omega }_{\mathrm{Ef}}$, where ${\alpha }_{\mathrm{Efb}}$ is the sum of the drag coefficients for the translating spherical cell body, ${\alpha }_{Cell}$, and filament ${\alpha }_{\mathrm{Ef}}$ (${\alpha }_{\mathrm{Efb}}={\alpha }_{Cell}+{\alpha }_{\mathrm{Ef}}$) and ${\beta }_{\mathrm{Ef}}$ is the drag coefficient for the flagellar rotation. Furthermore, ${v}_{\mathrm{Efb}}$ and ${\omega }_{\mathrm{Ef}}$ are the swimming speed and the flagellar rotation rate, respectively^27^. These drag coefficients depend on geometrical parameters such as the length and width of the cell body, the wavelength and the amplitude of a helix, and fluid viscosity (see “Methods”). Calculations using morphological parameters of *L. biflexa*^11^ and *E. coli* show that ${\alpha }_{\mathrm{Lep}}$ = − 0.056 pN·s/μm, ${\beta }_{\mathrm{Lep}}$ = 0.002 pN·s, ${\alpha }_{\mathrm{Ef}}$ = − 0.02 pN·s/μm, and ${\beta }_{\mathrm{Ef}}$ = 0.0003 pN·s. Thus, the drag coefficients for the leptospiral body are larger than those for *E. coli*. Despite these large drag coefficients, the swimming speed (~ 20 μm/s) and cell body rotation rates (> 100 Hz) of *Leptospira* spp. are comparable to those of *E. coli*^21^. Cryo-electron microscopy has showed that compared with conventional flagellar motors, such as *E. coli* and *Salmonella* spp., the rotor ring of the spirochetal flagellar motor is determined to be larger^28^, and a greater number of torque generators (stator units) are assembled^29^. Thus, the large swimming force of *Leptospira* could be provided by the high torque from a motor that rotates the heavy cell body. Since the cell morphology, motor structure, and swimming velocity are similar among different species of *Leptospira*^22,29^, the spirochetal genus is characterized as powerful swimmers.

The stall force was found to be uneven among measured cells (Fig. 4A). In the high load, the motor torque of the external flagellum depends on the number of stators and input energy ion motive force (IMF)^30^. Due to the stable incorporation of the full number of stator units to the motor, which is shown in *Leptospira* spp. by electron cryo-tomography^29^, the difference in IMF could be a cause of varied stall force. Furthermore, since intimate contact between PFs and cell membranes is necessary for spirochetal swimming^31^, PF-membrane interaction or morphological differences of PFs, such as length, could affect propulsion.

The force–speed relationship (Fig. 4B) showed that swimming force decreases with increased speed. *Leptospira* might have a mechanism to control propulsive output in response to changes in load. However, although the load-dependent assembly of stator units is observed in flagellar motors of *E. coli* and *Salmonella*^32–35^, these stator dynamics seem to be implausible for *Leptospira* motor as mentioned above. A motility study of *B. burgdorferi* by Harman et al. has discussed the balance between power input by the flagellar motor and power dissipation by swimming through a viscous liquid. According to Harman et al., the power input is defined by the motor torque ($M$) times the cell rotation rate $\omega$ and the power dissipation given by the sum of the term proportional to ${\omega }^{2}$ and proportional to $Fv$: $M\omega =A{\omega }^{2}+BFv$, where $A$ and $B$ are proportional coefficients comprising the parameters for cell morphology and drag coefficients^36^. Based on $v\propto \omega$^11,19^ and the assumption that $M$ is not dependent on load, the equation can be simplified into $F=C-Dv$, where $C$ and $D$ are proportional coefficients. Thus, the power dissipation model explains the observed force–speed relationship plausibly. Although the current experiments could not determine whether the speed-dependent reduction of swimming force is due to active force control by *Leptospira* or power dissipation by moving through viscous media, the spirochete is expected to invade highly viscous environments while maintaining a large output of power.

## Methods

### Bacteria and media

The saprophytes *Leptospira biflexa* strain Patoc I and *Leptospira kobayashii*^37^, and the pathogenic species *Leptospira interrogans* serovar Manilae strain UP-MMC-NIID^38^ were grown at 30°C for 4 days in Ellinghausen–McCullough–Johnson–Harris (EMJH) liquid medium with 10% bovine serum albumin until the late-exponential phase (Fig. S2). Potassium phosphate buffer (20 mM, pH 7.4) was used as a motility medium^12^.

### Measurement of swimming reversal

The *Leptospira* culture was centrifuged at 1,000*g* for 10 min at 23 °C, and the precipitated cells were resuspended in the motility medium without dilution. The bacterial suspension was infused to a flow chamber that was made by sticking a coverslip and a glass slide with double-sided tape (90 μm in thickness) that contained 1% agar so that the agar and liquid area were contiguous in the chamber (Fig. 2A). The liquid-agar border was observed with a dark-field microscope (BX53, 40× objective, 5× relay lens, Olympus, Tokyo, Japan), and behaviors of cells inserting one end of the cell body into agar were recorded with the CMOS camera at 250 Hz.

The swimming reversal was measured by tracing the cellular centroid in general^39,40^. However, the morphology of the *Leptospira* cell changes frequently, thus affecting the consistency between the centroid displacement and the actual cell movement (Supplementary Fig. S3). The positions of both ends of the cell body were determined together with the centroid, and simultaneous displacements of the three points were recognized as cell movements to avoid the false recognition of the reversal (Fig. S3A). Swimming speeds were measured by line fitting to the time courses of the cell displacements at an interval of 0.1 s, < 1 μm/s was judged as “pausing”. The reversal frequency was determined by normalizing the number of the reversals (${N}_{rev}$) by the observation time ($t$), such that ${N}_{rev}/t$. The data were analyzed with ImageJ software (National Institutes of Health, Rockville, MD) and programs originally developed using LabVIEW 2014 (National Instruments). Data of each condition were obtained by more than five independent experiments.

### Measurement of swimming force

A dark-field microscope (BX50, Olympus, Tokyo, Japan) that is essential for observing a thin leptospiral cell body (~ 140 nm in diameter) was equipped with an optical tweezer (Fig. 2A). The cell suspension prepared by the same procedure as the reversal measurement was mixed with 1.0 μm carboxyl latex beads (ThermoFisher Scientific, Waltham, MA) and was incubated at 23 °C for 10 min. The mixture was infused to the glass-made chamber, and spontaneous bead attachments to swimming cells were observed. The attached beads did not interfere with the *Leptospira* swimming (Video 7). The attached bead was trapped by a 1064 nm semiconductor laser (TLD001, Laser Diode Driver, Thorlabs Inc. Newton, NJ) through an ×100 oil immersion objective lens (UPlanFLN, Olympus, Tokyo, Japan), and the bead movement was recorded with a CMOS video camera (acA800-510um, Basler, Ahrensburg, Germany) at a frame rate of 500 Hz. The numerical aperture of the objective was adjusted with the objective-lens aperture to perform dark-field observation and laser trapping simultaneously. The recorded movie was analyzed to determine the bead displacement with a custom-made program developed using LabVIEW 2014 (National Instruments, Austin, TX).

Displacement of a trapped bead ($\Delta x$) can be calibrated to a restoring force of optical tweezer (${F}_{trap}$) using the equation ${F}_{trap}=k\Delta x$, where $k$ is a spring constant. The values of $k$ were determined in each flow chamber by trapping a bead free from cells and analyzing its positional fluctuation. The positional distribution of the trapped bead showed a Gaussian distribution $f\left(x\right)\propto exp\left(-{x}^{2}/2{\sigma }^{2}\right)$, where $\sigma$ is the standard deviation (Fig. 2B, black line), obeying Boltzmann’s law $P\left(x\right)\propto exp\left(-\Delta U/{k}_{B}T\right)$, where $\Delta U$ is the potential energy, ${k}_{B}$ is the Boltzmann constant (1.38 ×10^-23^ J/K), and $T$ is the absolute temperature (296 K), namely, $\Delta U=\left( {k}_{B}T/2{\sigma }^{2}\right){x}^{2}$ (Fig. 2B, red line). Since the thermal fluctuation of the bead captured by a spring with $k$ can be described by the harmonic function $U\left(x\right)=1/2k{x}^{2}$, $k={k}_{B}T/{\sigma }^{2}$. The swimming force was determined using ${F}_{trap}$ and ${F}_{drag}$ (Fig. 2C). When the bead with a diameter of $r$ is moved at a speed of $v$ (swimming speed of the cell) in a solution with a viscosity of $\mu$, ${F}_{drag}=6\pi \mu rv$, where $6\pi \mu r$ is a drag coefficient given by Stokes’ low. The viscosity of the motility medium was measured with a tuning-fork-type viscometer (SV-1A, A&D, Tokyo, Japan), giving 0.8 mPa s at 23 °C. Data were obtained by five independent experiments.

### Drag coefficients

Drag coefficients for the spirochete and externally flagellated bacterium are calculated as described previsouly^11,27^. For the *Leptospira* cell, the protoplasmic cylinder lacking bending at the cell ends was assumed:

$${\alpha }_{\mathrm{Lep}}={C}_{Lep}\left(8{\pi }^{2}{{r}_{Lep}}^{2}+{{p}_{Lep}}^{2}\right);$$

$${\beta }_{\mathrm{Lep}}={-2C}_{Lep}\pi {{r}_{Lep}}^{2}{p}_{Lep};$$

$${C}_{Lep}=2\pi \mu {L}_{Lep}/\left\{\left(\mathrm{log}[{d}_{Lep}/2{p}_{Lep}]+0.5\right)\left(4{\pi }^{2}{{r}_{Lep}}^{2}+{{p}_{Lep}}^{2}\right)\right\}.$$

Here, ${r}_{Lep}$, ${p}_{Lep}$, ${L}_{Lep}$, and ${2d}_{Lep}$ are the helix radius (0.09 μm), helix pitch (0.7 μm), length (20 μm), and diameter (0.14 μm) of the leptospiral cell body^11,41^. Drag coefficients for the externally flagellated bacterium that was assumed to consist of a spherical body and a helical filament, referring to the morphology of *Vibrio alginolyticus*^27^, were calculated as follows:

$${\alpha }_{Cell}=-6\pi \mu a\left\{1-0.2\left(1-b/a\right)\right\};$$

$${\alpha }_{\mathrm{Ef}}={C}_{Ef}\left(8{\pi }^{2}{{r}_{Ef}}^{2}+{{p}_{Ef}}^{2}\right);$$

$${\beta }_{\mathrm{Ef}}={-2C}_{Ef}\pi {r}^{2}{p}_{Ef};$$

$${C}_{Ef}=2\pi \mu {L}_{Ef}/\left\{\left(\mathrm{log}[{d}_{Ef}/2{p}_{Ef}]+0.5\right)\left(4{\pi }^{2}{{r}_{Ef}}^{2}+{{p}_{Ef}}^{2}\right)\right\}.$$

Here, $2a$ and $2b$ are the diameter (0.8 μm) and length (1.92 μm) of the cell body, and ${r}_{Ef}$, ${p}_{Ef}$, ${L}_{Ef}$, and $2{d}_{Ef}$ are the helix radius (0.14 μm), helix pitch (1.58 μm), length (5.02 μm), and diameter (0.032 μm) of the flagellum. The medium viscosity $\mu$ was assumed to be 1 mPa s.

## Supplementary information

Supplementary Information.

Supplementary Video 1.

Supplementary Video 2.

Supplementary Video 3.

Supplementary Video 4.

Supplementary Video 5.

Supplementary Video 6.

Supplementary Video 7.

## Data Availability

The data supporting the findings of this study are available from the corresponding author upon request.
