# Supplementary material for: Implications of back-and-forth motion and powerful propulsion for spirochetal invasion
Source: Sci Rep. 2020 Aug 18;10:13937. doi: 10.1038/s41598-020-70897-z (PMC7434897; doi:10.1038/s41598-020-70897-z)
Supplement: Supplementary file 1 — Supplementary Information. [file 41598_2020_70897_MOESM1_ESM.pdf]

Supplementary Information

Title: Implications of back-and-forth motion and powerful propulsion for spirochetal invasion

Authors: K. Abe, T. Kuribayashi, K. Takabe, and S. Nakamura

· Supplementary Fig. S1. Swimming force vs time plots.
· Supplementary Fig. S2. Growth curve of *L. kobayashii*.
· Supplementary Fig. S3. Swimming reversal measurement.
· Supplementary Video 1. Trial-and-error and invasion of *L. kobayashii* at the liquid-agar border
· Supplementary Video 2. Swimming reversal of *L. interrogans* at the liquid-agar border
· Supplementary Video 3. Swimming reversal of *L. biflexa* at the liquid-agar border
· Supplementary Video 4. Movement of a bead on the cell body of *L. kobayashii*
· Supplementary Video 5. Laser trap of 1-μm polystyrene beads for force measurement
· Supplementary Video 6. Quick swimming reversal of *L. kobayashii* (slow-motion movie)
· Supplementary Video 7. Untrapped *L. kobayashii* attached with a bead

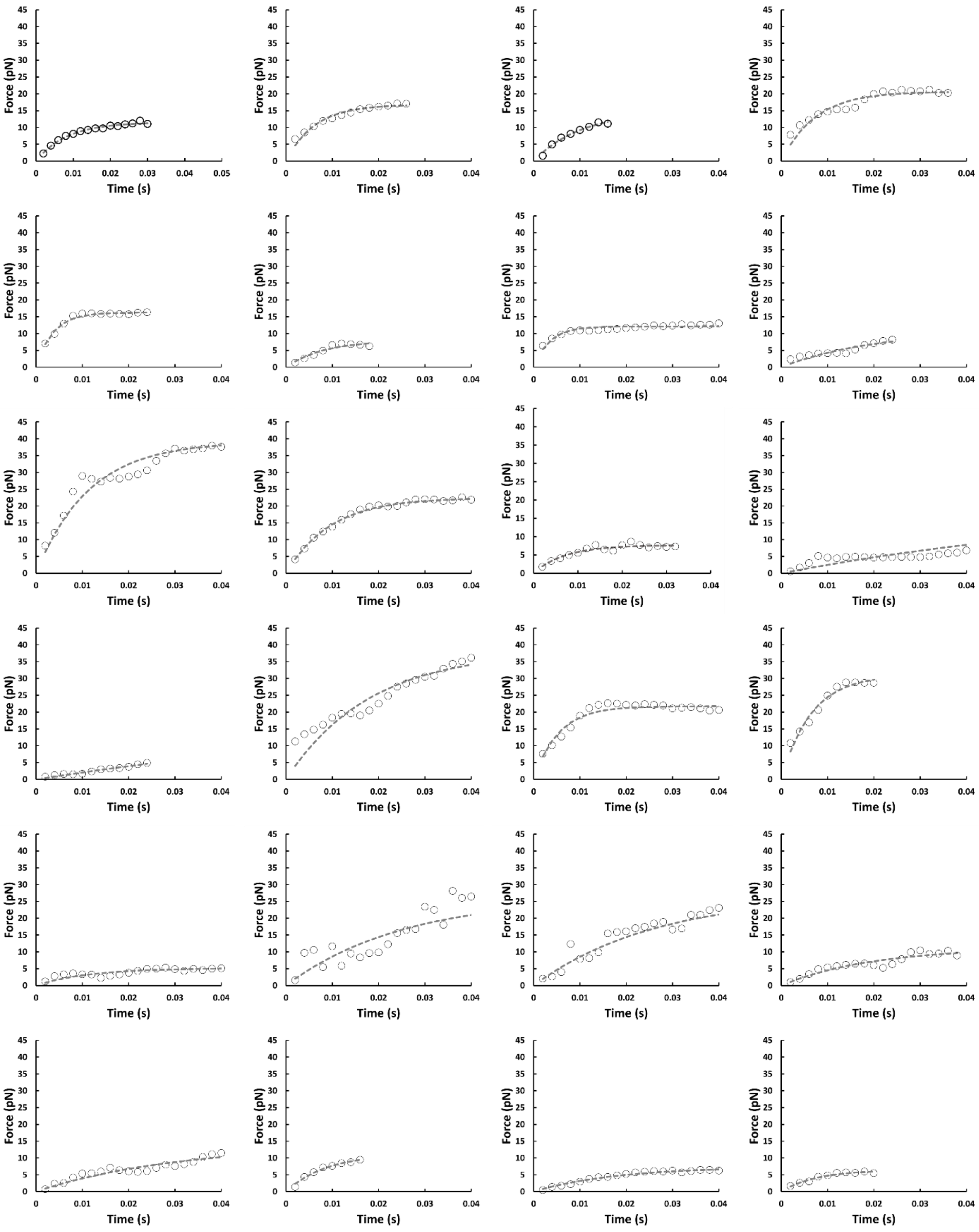

Supplementary Fig. S1. Swimming force vs time plots. Circles are experimental data plot, and dashed lines are the results of exponential curve fitting.

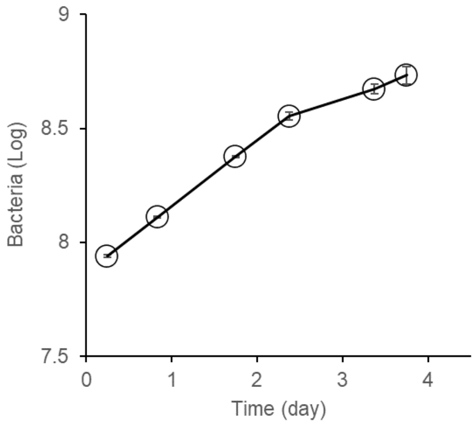

Supplementary Fig. S2. Growth curve of *L. kobayashii*. The average values and standard deviations (error bars) obtained from three independent measurements are shown. At the 4th day after starting cultivation, the growth rate gets slow, but does not reach a stationary phase. Therefore, we defined the growth phase of bacteria used for motility assay as the late-exponential phase.

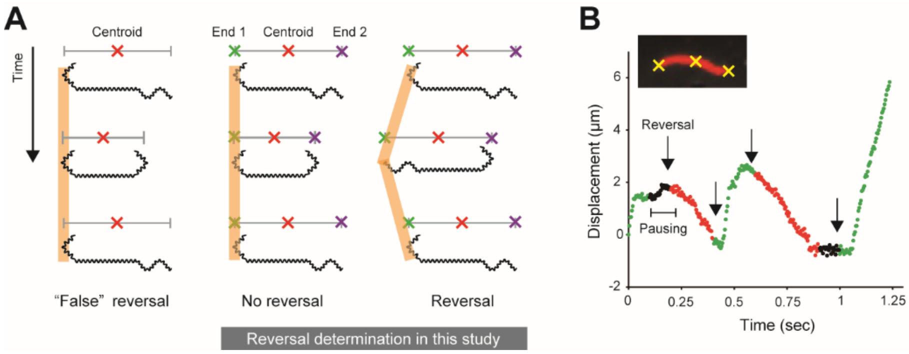

Supplementary Fig. S3. Swimming reversal measurement. (A) Determination of swimming reversal. The left panel shows a conventional measurement where the cellular centroids are traced; the center and right panels explain the current method, in which the positions of both cellular ends are determined together with the centroid. Thick orange lines indicate the actual displacement. See Materials and Methods for detailed explanation. (B) Example data of the leptospiral displacement. Green, red, and black indicate the forward movement, backward movement, and pausing, respectively. This data shows four reversals (black arrows). The inset shows the analyzed microscopic image of a leptospiral cell.
